# Unfractionated heparin may improve near-term survival in patients admitted to the ICU with sepsis attributed to pneumonia: an observational study using the MIMIC-IV database

**DOI:** 10.3389/fphar.2025.1518716

**Published:** 2025-02-27

**Authors:** Shusheng Fang, Yali Wang, Wenyu Nan, Yanhao Feng, Wen Su, Yiming Wang, Xiaodong Jiang

**Affiliations:** The Second Hospital of Dalian Medical University, Dalian Medical University, Dalian, Liaoning, China

**Keywords:** unfractionated heparin, pneumonia, sepsis, short-term survival, MIMIC-IV database

## Abstract

**Introduction:**

Limited data are available on the use, duration, and dosage of anticoagulant therapy in patients with pneumonia-induced sepsis, and the survival benefits of heparin remain uncertain. This study aimed to assess whether heparin administration improves near-term survival in critically ill patients with pneumonia-induced sepsis and identify the optimal dosage and treatment duration.

**Methods:**

This study utilized the Medical Information Mart for Intensive Care-IV (MIMIC-IV) database. The variance inflation factor was employed to exclude highly collinear variables. Propensity score matching (PSM), the Cox proportional hazards model, and Cox regression subgroup analysis were used to evaluate the outcomes of subcutaneous heparin prophylactic anticoagulation after intensive care unit (ICU) admission. The primary outcomes were 30-, 45-, and 60-d mortality rates. Secondary outcomes included ICU length of stay (LOS_ICU), hospital length of stay (LOS_Hospital), in-hospital mortality, and the incidence of gastrointestinal bleeding.

**Results:**

We enrolled 1,586 adult patients with pneumonia-induced sepsis. After PSM, 1,176 patients remained (588 in the heparin group and 588 in the non-heparin group). The 45-d survival rate was significantly higher in the heparin-treated group than that in the non-heparin group (84.4% vs. 79.4%; HR: 0.75; 95% CI: 0.572–0.83; adjusted HR: 0.73, 95% CI: 0.563–0.964; P < 0.05). LOS_ICU and LOS_Hospital were significantly shorter in the heparin group (P < 0.001), with no significant difference in gastrointestinal bleeding incidence between the two groups. Cox proportional hazards models demonstrated that heparin dose and duration were strongly associated with 45-d survival. Subgroup analysis indicated a significant survival advantage in patients aged 18–60 years, without diabetes, chronic obstructive pulmonary disease, or stage 1 acute kidney injury, who received a daily heparin dose of 3 mL for more than 7 d.

**Conclusion:**

Our study found that early administration of heparin, particularly in sufficient doses (Heparin Sodium 5,000 units/mL, 1 mL per dose, three times daily (TID)) for more than 7 d, was associated with reduced near-term mortality in critically ill patients with pneumonia-induced sepsis. These findings underscore the potential benefits of anticoagulant therapy in this high-risk patient population.

## 1 Introduction

Pneumonia, an infection of the lung parenchyma, encompasses a group of syndromes caused by various pathogens, each presenting with different manifestations and complications (Mackenzie, 2016). It remains a common and burdensome disease across all populations. A study by the Centers for Disease Control and Prevention (CDC) estimated that pneumonia is the eighth leading cause of death in the United States after adjusting for sex and age differences (Jain et al., 2015). Despite its severity, pneumonia is often overlooked due to low public awareness, insufficient economic investment from both public and private sectors, and inadequate advocacy (Aliberti et al., 2019). Consequently, it frequently leads to intensive care unit (ICU) admission and progresses to sepsis (Zaragoza et al., 2020).

Pneumonia is the most common cause of sepsis and septic shock—life-threatening conditions resulting from a dysregulated host response to infection (Gauer et al., 2020). Since 2017, an estimated 48.9 million sepsis cases and 11 million sepsis-related deaths have been reported globally, accounting for 19.7% of all deaths worldwide. Despite advancements in medical technology that have reduced sepsis incidence and mortality, it remains the leading cause of health loss worldwide (Rudd et al., 2020). The pathogenesis of sepsis involves systemic inflammation and coagulation upregulation, which are key host defense mechanisms against invading pathogens (Aird, 2003; Iba and Levy, 2020). Clotting system activation is common in sepsis; however, excessive activation can lead to disseminated intravascular coagulation (DIC), resulting in organ dysfunction and fatal outcomes (Iba and Levy, 2020).

Given the close relationship between inflammation and coagulation in sepsis, anticoagulants have been considered as potential therapeutic agents to mitigate the harmful effects of excessive clotting (Vagionas et al., 2022). Heparin, a naturally occurring polysaccharide, is known for its anticoagulant and anti-inflammatory properties, which are independent of its effects on coagulation (Anastase-Ravion et al., 2003; Yini et al., 2015). It has been shown to reduce inflammation by inhibiting key inflammatory mediators, such as caspase-11, and its anticoagulant properties help prevent microvascular thrombosis—a common complication in sepsis. Additionally, heparin has demonstrated protective effects by reducing endothelial injury, enhancing vascular integrity, and improving organ function (Cornet et al., 2007; Li and Ma, 2017; Tang et al., 2021; Zarychanski et al., 2015).

Pneumonia-induced sepsis arises from an imbalance between pathogenic organisms in the lower respiratory tract and both local and systemic immune defenses (Torres et al., 2021). It can lead to severe complications, such as acute kidney injury (AKI) and DIC, significantly increasing patient mortality (Kim et al., 2013). However, the efficacy and safety of heparin in pneumonia-related sepsis remain controversial. To evaluate the impact of heparin use in these patients, we utilized the Medical Information Mart for Intensive Care IV (MIMIC-IV) database, excluding conditions with significant prognostic impact that require anticoagulation. Our goal was to determine whether heparin administration following ICU admission is associated with reduced near-term mortality in patients with pneumonia-induced sepsis.

## 2 Materials and methods

### 2.1 Data source: MIMIC-IV

The MIMIC-IV database (https://physionet.org/content/mimiciv/3.0/) is a single-center, open-access database containing 94,458 patients admitted to the ICU at the Beth Israel Deaconess Medical Center in the United States between 2008 and 2022 (Johnson et al., 2023). Shusheng Fang collected clinical data from the MIMIC-IV database (certification number: 63162441), including patient demographic information, laboratory findings, and medications. The use of the database was approved by the Institutional Review Boards of the Massachusetts Institute of Technology and Beth Israel Deaconess Medical Center. This project complied with the Declaration of Helsinki; ethical approval was not required because of participant anonymity and data standardization in this database. All methods were performed in accordance with relevant guidelines and regulations.

### 2.2 Inclusion and exclusion criteria

Patients were considered eligible if they were diagnosed with pneumonia using the ICD-9 (4829, 99731, 48242, 48241, 4870, 481,4820, 0382, 4809, 99732, 485, 48881, 4830, 48249, 48801, 51637, 11505, 51635, 48811) and ICD-10 (B961, J95851, J159, J1289, J188, J13, J150, J181, J1008, J851, J1000, J129, J1100, A403, J21, J1108, J180, J09X1, J122, J1001, J8281, J123, J157, B960, J4811, J8282, J120, J84117, J160, J842, A0222, J182, B012, Z8701, V1261) codes after their first ICU admission. The inclusion criteria were (Mackenzie, 2016) aged ≥18 years (Jain et al., 2015); suspected sepsis occurring after or at the same time as pneumonia diagnosis (Aliberti et al., 2019); meeting the sepsis-3 diagnostic criteria (Rhodes et al., 2016): infection + Sequential Organ Failure Assessment (SOFA) score ≥2.

The exclusion criteria were patients (Mackenzie, 2016) diagnosed with cryptogenic pneumonia, tuberculous pneumonia, postoperative aspiration pneumonia, or preventive inoculation pneumonia vaccine (Jain et al., 2015); who had multiple admissions other than the first and stayed in the hospital or ICU for less than 24 h (Aliberti et al., 2019); who had malignant tumors, metastatic solid tumors, or acquired immunodeficiency syndrome (Zaragoza et al., 2020); requiring anticoagulation due to conditions causing coagulopathy, such as acute myocardial infarction, arterial occlusion, arterial thrombosis, venous thrombosis, pulmonary embolism, cerebral infarction, and cirrhosis; and (Gauer et al., 2020) who received heparin intravenously.

Patients with pneumonia-induced sepsis who were first admitted to the ICU were divided into two groups: those who received heparin and those who did not. Quality control measures were implemented to ensure accuracy and consistency, particularly for key variables, such as the number of hospitalizations, diagnostic codes, and medication management records. After the initial screening, instances of multiple hospital admissions for the same patient were addressed by retaining only the first admission and the first ICU admission record. For the use of heparin, the database contained records of heparin being used for different purposes. To ensure proper inclusion, we cross-referenced the database to confirm that only heparin used for injection into the body had a pharmacy_id of 86768272.

### 2.3 Data collection

Structured query language was used to obtain patient information from MIMIC-IV 3.0 in Navigate Premium (version 16). The code was obtained from https://github.com/MIT-LCP/ mimic-iv/concepts_postgres. Patient demographics, including age, sex, and race, were obtained. Vital signs, including heart rate, respiratory rate, mean arterial pressure (MAP), and oxygen saturation (SpO₂), were measured upon patient admission. The following comorbidities were recorded: hypertension, diabetes, AKI, acute respiratory failure (ARF), and chronic obstructive pulmonary disease (COPD).

Additionally, the initial laboratory indicators of the patient on the first day of admission were extracted, including the count of red blood cells (RBCs), white blood cells (WBCs), platelet (PLT), hemoglobin, hematocrit, red blood cell distribution width (RDW), calcium, chloride, sodium, potassium, bicarbonate, anion gap, creatinine, glucose, blood urea nitrogen (BUN), international normalized ratio (INR), prothrombin time (PT), partial thromboplastin time (PTT), pH value (PH), arterial partial pressure of oxygen (PO₂), arterial partial pressure of carbon dioxide (PCO₂), lactate, and urine output.

The Acute Physiology Score III (APS III), Glasgow Coma Scale (GCS), and Charlson Comorbidity Index (CCI) were calculated upon ICU admission, along with SOFA scores after suspected sepsis. The following treatment information was noted: the use of mechanical ventilation, vasopressors, and unfractionated heparin (UFH) (Heparin Sodium 5,000 units/mL - 1 mL vial).

### 2.4 Primary outcome and secondary outcomes

The primary outcomes of this study were the 30-, 45-, and 60-d mortality rates. Secondary outcomes included ICU stay duration (LOS_ICU), hospital stay duration (LOS_Hospital), in-hospital mortality, and gastrointestinal hemorrhage.

### 2.5 Statistical analysis

As this study was a retrospective analysis, no sample size calculations were performed. All variables had less than 15% missing values (Supplementary Table S1). The random forest imputation method was employed, specifically using the multiple imputation functionality in the mice package in R version 4.2.3 (with m = 5 to generate five imputed datasets and maxit = 50 to ensure convergence of the imputation process). Missing values were imputed using the random forest algorithm. To improve the stability of the imputation results, a mean merging approach was applied, where numeric variables from the five imputed datasets were summed and averaged to generate the final imputed dataset.

The variance inflation factor (VIF) was used to assess multicollinearity among variables. Some variables with a VIF exceeding five were removed because of multicollinearity concerns. Patients treated with heparin during hospitalization constituted the experimental group, while those not treated with heparin were the control group. The Mann–Whitney U test was used for non-normally distributed continuous variables, which were expressed as medians and interquartile ranges. Categorical variables for the two groups were expressed as numbers with proportions and compared using the chi-square test. All data were analyzed using RStudio (version 4.2.3), with statistical significance defined as P ≤ 0.05.

Propensity score matching (PSM) (caliper value, 0.05) was used to narrow the gap in baseline characteristics between the two groups. Kaplan–Meier curves were used to indicate the occurrence of 30-, 45-, and 60-d mortality in patients with sepsis. Cox proportional hazard models were used to estimate the association between mortality and patients who received or did not receive heparin. Additionally, patients with sepsis were divided into different subgroups based on age, sex, hypertension, diabetes, ARF, COPD, stage of AKI, mechanical ventilation, and vasoactive drug use, which were analyzed separately for hazard ratios (HR) and 95% confidence intervals (CI).

## 3 Results

### 3.1 Patient characteristics

**FIGURE 1:**
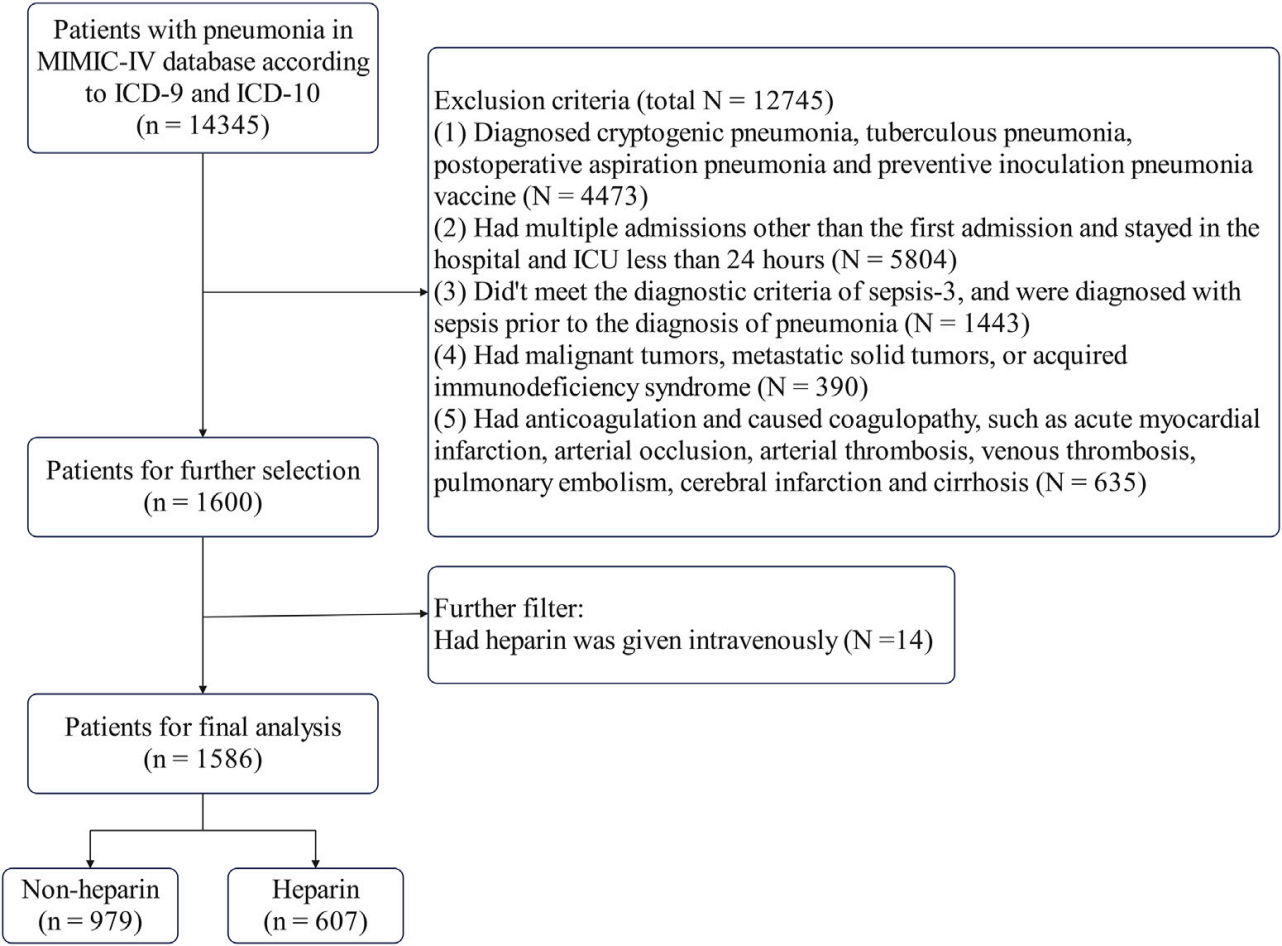
Flow diagram of the research.

Figure 1 shows the patient selection procedure. Supplementary Table S2 presents the VIFs of the data variables, showing that RBC, hemoglobin, hematocrit, chloride, and anion gap had VIFs >5. Due to multicollinearity, hemoglobin, hematocrit, and chloride were removed. After removing these variables, Supplementary Table S3 shows that the VIFs of the remaining variables were all below 5 (Supplementary Tables S2, S3).

A total of 1,586 patients met the inclusion criteria, with 607 receiving heparin. As this was a retrospective study, the results were susceptible to confounding factors. PSM was used to minimize the effect of confounding factors using propensity scores to match individuals in the control group with those in the experimental group who had similar background characteristics and disease severity (age, lactic acid level, and SOFA score). Data from 1586 patients were extracted from the MIMIC-IV database. PSM matched 1176 patients (588 in the control and 588 in the experimental groups). After PSM, the influence of confounding factors was minimized, making the primary distinguishing factor the presence or absence of heparin.

Tables 1, 2 present the baseline characteristics of the patients in both groups before and after PSM. Before PSM, the patients in the control group were older than those in the experimental group. Patients who did not use heparin had a higher prevalence of AKI and hypertension and required mechanical ventilation more frequently. Significant differences were observed in the CCI score, GCS score, respiratory rate, SpO₂, PLT, calcium, BUN, pH, PO₂, PCO₂, lactic acid, urine volume, INR, and PT (P < 0.05) (Table 1).

**TABLE 1:** Baseline characteristics of the included patients after PSM.

		Overall	Non-heparin	Heparin	p
		(n = 1586)	(n = 979)	(n = 607)	
Age (years)		65.65 [53.50, 77.35]	66.74 [54.72, 77.39]	63.15 [50.96, 77.29]	0.032
Gender (%)					0.115
	Female	670 (42.2)	398 (40.7)	272 (44.8)	
	Male	916 (57.8)	581 (59.3)	335 (55.2)	
Race (%)					0.542
	Black	179 (11.3)	106 (10.8)	73 (12.0)	
	White	872 (55.0)	530 (54.1)	342 (56.3)	
	Other	207 (13.1)	134 (13.7)	73 (12.0)	
	Unknown	328 (20.7)	209 (21.3)	119 (19.6)	
Charlson Comorbidity Index score		5.00 [3.00, 7.00]	5.00 [3.00, 7.00]	5.00 [3.00, 7.00]	0.001
SOFA score		3.00 [2.00, 5.00]	3.00 [2.00, 5.00]	3.00 [2.00, 4.00]	0.838
GCS score		10.00 [6.00, 14.00]	10.00 [5.00, 14.00]	11.00 [6.00, 14.00]	0.006
APSIII score		60.00 [44.00, 82.00]	60.00 [44.00, 81.00]	61.00 [45.00, 83.50]	0.32
Mechanical Ventilation (%)					0.049
	No	491 (31.0)	285 (29.1)	206 (33.9)	
	Yes	1095 (69.0)	694 (70.9)	401 (66.1)	
Norepinephrine (%)					0.755
	No	806 (50.8)	494 (50.5)	312 (51.4)	
	Yes	780 (49.2)	485 (49.5)	295 (48.6)	
ARF (%)					0.363
	No	651 (41.0)	411 (42.0)	240 (39.5)	
	Yes	935 (59.0)	568 (58.0)	367 (60.5)	
COPD (%)					0.093
	No	1454 (91.7)	907 (92.6)	547 (90.1)	
	Yes	132 (8.3)	72 (7.4)	60 (9.9)	
AKI Stage (%)					0.004
	Non-AKI	239 (15.1)	132 (13.5)	107 (17.6)	
	AKI-1	997 (62.9)	644 (65.8)	353 (58.2)	
	AKI-2	319 (20.1)	180 (18.4)	139 (22.9)	
	AKI-3	31 (2.0)	23 (2.3)	8 (1.3)	
Hypertension (%)					0.009
	No	926 (58.4)	546 (55.8)	380 (62.6)	
	Yes	660 (41.6)	433 (44.2)	227 (37.4)	
Diabetes (%)					0.258
	No	1050 (66.2)	659 (67.3)	391 (64.4)	
	Yes	536 (33.8)	320 (32.7)	216 (35.6)	
Respiratory Rate (breaths/min)		20.00 [16.00, 24.00]	19.00 [16.00, 24.00]	20.00 [16.00, 25.00]	0.002
Heartrate (beats/min)		88.00 [75.00, 105.00]	88.00 [75.00, 104.00]	89.00 [75.00, 107.00]	0.421
MAP (mmHg)		81.50 [70.00, 95.00]	82.00 [70.00, 96.00]	81.00 [70.00, 93.00]	0.255
SpO2 (%)		98.00 [94.00, 100.00]	98.00 [95.00, 100.00]	97.00 [94.00, 100.00]	0.002
RBC (10^9/L)		3.67 [3.15, 4.23]	3.67 [3.11, 4.24]	3.68 [3.25, 4.21]	0.25
WBC(10^9/L)		11.70 [8.40, 16.48]	11.80 [8.80, 16.40]	11.30 [7.70, 16.70]	0.236
PLT (10^9/L)		200.00 [147.00, 266.00]	195.00 [144.00, 261.50]	206.00 [152.50, 275.00]	0.004
RDW (%)		14.30 [13.40, 15.70]	14.20 [13.40, 15.70]	14.40 [13.45, 15.70]	0.163
Sodium (mmol/L)		139.00 [136.00, 142.00]	139.00 [136.00, 142.00]	139.00 [136.00, 142.00]	0.89
Potassium (mmol/L)		4.10 [3.70, 4.60]	4.20 [3.70, 4.60]	4.10 [3.70, 4.60]	0.637
Calcium (mg/dL)		8.27 [7.70, 8.80]	8.30 [7.80, 8.80]	8.10 [7.60, 8.70]	<0.001
BUN (mg/dL)		21.00 [14.00, 35.00]	21.00 [14.00, 33.00]	22.00 [14.00, 37.00]	0.06
Creatinine (mg/dL)		1.00 [0.72, 1.60]	1.00 [0.80, 1.50]	1.10 [0.70, 1.80]	0.169
Glucose (mg/dL)		137.00 [111.00, 179.75]	136.00 [112.00, 180.50]	137.00 [110.00, 178.00]	0.757
PH		7.36 [7.29, 7.42]	7.37 [7.30, 7.42]	7.35 [7.28, 7.41]	0.001
PO2 (mmHg)		101.00 [64.00, 175.00]	112.00 [70.00, 201.00]	88.00 [59.00, 140.00]	<0.001
PCO2 (mmHg)		42.00 [36.00, 50.00]	41.00 [36.00, 49.00]	43.00 [37.00, 51.00]	<0.001
Anion Gap (mmol/L)		14.00 [12.00, 17.00]	14.00 [12.00, 17.00]	14.00 [12.00, 17.00]	0.932
Bicarbonate (mmol/L)		23.00 [20.00, 26.00]	23.00 [20.00, 25.00]	23.00 [19.00, 26.00]	0.945
Lactate (mmol/L)		1.64 [1.20, 2.42]	1.70 [1.20, 2.50]	1.60 [1.10, 2.40]	0.016
Urine Output (mL)		1535.00 [915.00, 2412.50]	1595.00 [956.50, 2476.00]	1450.00 [875.00, 2285.00]	0.022
INR		1.30 [1.10, 1.50]	1.30 [1.10, 1.57]	1.20 [1.10, 1.40]	<0.001
PT (s)		13.90 [12.50, 16.10]	14.20 [12.60, 17.00]	13.50 [12.40, 15.00]	<0.001
PTT (s)		31.00 [27.50, 37.10]	31.20 [27.30, 38.40]	30.80 [27.80, 35.40]	0.129

**TABLE 2:** Baseline characteristics of the included patients after PSM.

		Overall	Non-heparin	Heparin	p
		(n = 1176)	(n = 588)	(n = 588)	
Age (years)		63.90 [51.99, 76.56]	64.77 [51.99, 76.16]	63.24 [52.01, 77.09]	0.897
Gender (%)					0.481
	Female	515 (43.8)	251 (42.7)	264 (44.9)	
	Male	661 (56.2)	337 (57.3)	324 (55.1)	
Race (%)					0.32
	Black	133 (11.3)	63 (10.7)	70 (11.9)	
	White	645 (54.8)	311 (52.9)	334 (56.8)	
	Other	156 (13.3)	85 (14.5)	71 (12.1)	
	Unknown	242 (20.6)	129 (21.9)	113 (19.2)	
Charlson Comorbidity Index score		5.00 [3.00, 7.00]	5.00 [3.00, 7.00]	5.00 [3.00, 7.00]	0.082
SOFA score		3.00 [2.00, 4.00]	3.00 [2.00, 4.00]	3.00 [2.00, 4.00]	0.908
GCS score		10.00 [6.00, 14.00]	9.00 [5.00, 14.00]	11.00 [6.00, 14.00]	0.001
APSIII score		60.50 [44.00, 82.00]	61.00 [44.00, 82.00]	60.00 [44.75, 82.00]	0.612
Mechanical Ventilation (%)					0.052
	No	372 (31.6)	170 (28.9)	202 (34.4)	
	Yes	804 (68.4)	418 (71.1)	386 (65.6)	
Norepinephrine (%)					0.382
	No	594 (50.5)	289 (49.1)	305 (51.9)	
	Yes	582 (49.5)	299 (50.9)	283 (48.1)	
ARF (%)					0.858
	No	468 (39.8)	232 (39.5)	236 (40.1)	
	Yes	708 (60.2)	356 (60.5)	352 (59.9)	
COPD (%)					0.149
	No	1073 (91.2)	544 (92.5)	529 (90.0)	
	Yes	103 (8.8)	44 (7.5)	59 (10.0)	
AKI Stage (%)					0.049
	Non-AKI	182 (15.5)	79 (13.4)	103 (17.5)	
	AKI-1	730 (62.1)	386 (65.6)	344 (58.5)	
	AKI-2	245 (20.8)	112 (19.0)	133 (22.6)	
	AKI-3	19 (1.6)	11 (1.9)	8 (1.4)	
Hypertension (%)					0.212
	No	704 (59.9)	341 (58.0)	363 (61.7)	
	Yes	472 (40.1)	247 (42.0)	225 (38.3)	
Diabetes (%)					0.157
	No	776 (66.0)	400 (68.0)	376 (63.9)	
	Yes	400 (34.0)	188 (32.0)	212 (36.1)	
Respiratory Rate (breaths/min)		20.00 [16.00, 24.00]	19.00 [16.00, 23.00]	20.00 [16.00, 25.00]	0.001
Heartrate (beats/min)		89.00 [75.00, 105.00]	88.00 [75.00, 104.00]	89.00 [75.00, 107.00]	0.494
MAP (mmHg)		82.00 [70.00, 95.00]	83.00 [70.00, 97.00]	81.00 [70.00, 93.00]	0.133
SpO2 (%)		97.00 [94.00, 100.00]	98.00 [95.00, 100.00]	97.00 [94.00, 100.00]	0.018
RBC (10^9^/L)		3.68 [3.18, 4.23]	3.74 [3.14, 4.26]	3.67 [3.25, 4.20]	0.784
WBC (10^9^/L)		11.70 [8.40, 16.50]	11.90 [9.00, 16.50]	11.30 [7.70, 16.70]	0.121
PLT (10^9^/L)		204.50 [149.75, 270.25]	199.00 [147.00, 268.00]	206.00 [152.75, 275.25]	0.079
RDW (%)		14.30 [13.40, 15.60]	14.10 [13.40, 15.60]	14.40 [13.50, 15.70]	0.137
Sodium (mmol/L)		139.00 [136.00, 142.00]	139.00 [136.00, 142.00]	139.00 [136.00, 142.00]	0.541
Potassium (mmol/L)		4.10 [3.70, 4.60]	4.15 [3.70, 4.60]	4.10 [3.70, 4.60]	0.717
Calcium (mg/dL)		8.20 [7.70, 8.70]	8.30 [7.80, 8.80]	8.10 [7.60, 8.70]	<0.001
BUN (mg/dL)		21.00 [14.00, 36.00]	20.00 [13.00, 33.00]	22.00 [14.00, 37.00]	0.06
Creatinine (mg/dl)		1.00 [0.80, 1.70]	1.00 [0.80, 1.50]	1.10 [0.70, 1.80]	0.103
Glucose (mg/dL)		136.50 [110.00, 179.00]	134.50 [111.00, 179.25]	137.50 [110.00, 178.00]	0.939
PH		7.36 [7.29, 7.42]	7.37 [7.30, 7.42]	7.35 [7.28, 7.41]	0.007
PO2 (mmHg)		96.50 [61.00, 166.25]	109.00 [68.00, 200.10]	87.00 [58.00, 139.10]	<0.001
PCO2 (mmHg)		42.10 [37.00, 50.65]	42.00 [36.00, 50.00]	43.30 [37.00, 51.00]	0.023
Anion Gap (mmol/L)		14.00 [12.00, 17.00]	14.00 [12.00, 17.00]	14.00 [12.00, 17.00]	0.975
Bicarbonate (mmol/L)		23.00 [20.00, 26.00]	23.00 [20.00, 26.00]	23.00 [20.00, 26.00]	0.916
Lactate (mmol/L)		1.60 [1.20, 2.40]	1.60 [1.20, 2.40]	1.60 [1.10, 2.30]	0.165
Urine Output (mL)		1532.50 [908.75, 2450.00]	1597.50 [939.25, 2554.75]	1450.50 [880.00, 2266.25]	0.016
INR		1.20 [1.10, 1.40]	1.30 [1.10, 1.50]	1.20 [1.10, 1.40]	<0.001
PT (s)		13.70 [12.40, 15.60]	13.99 [12.50, 16.70]	13.48 [12.40, 15.00]	<0.001
PTT (s)		30.92 [27.43, 36.50]	31.16 [27.17, 38.37]	30.80 [27.70, 35.32]	0.257

PSM with a caliper value of 0.05 was applied to reduce the influence of confounding variables and improve comparability between the experimental and control groups. After PSM, the control group still had a higher prevalence of AKI, with statistically significant differences in GCS, respiratory rate, SpO₂, calcium, pH, PO₂, PCO₂, urine volume, INR, and PT than the experimental group (P < 0.05) (Table 2). The results confirm that the baseline characteristics of the two groups were well balanced after PSM, ensuring the reliability of the subsequent analysis.

### 3.2 Association between heparin and mortality outcomes

**FIGURE 2:**
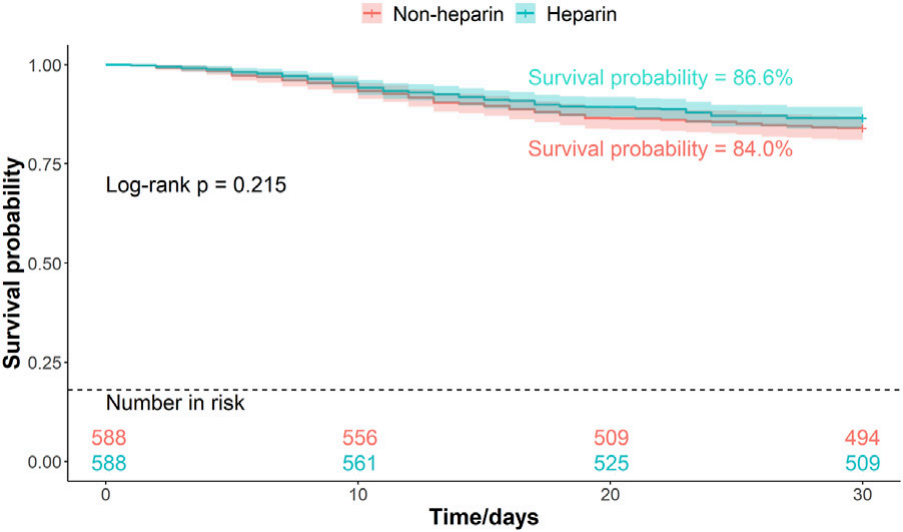
Kaplan-Meier survival curves between the two groups indicating 30-day mortality risk in patients with pneumonia sepsis. (After PSM).

**FIGURE 3:**
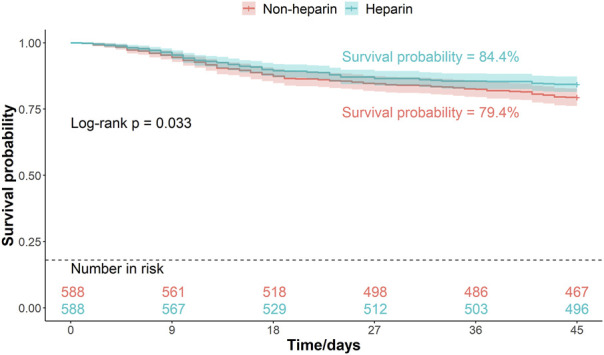
Kaplan-Meier survival curves between the two groups indicating 45-day mortality risk in patients with pneumonia sepsis. (After PSM).

**FIGURE 4:**
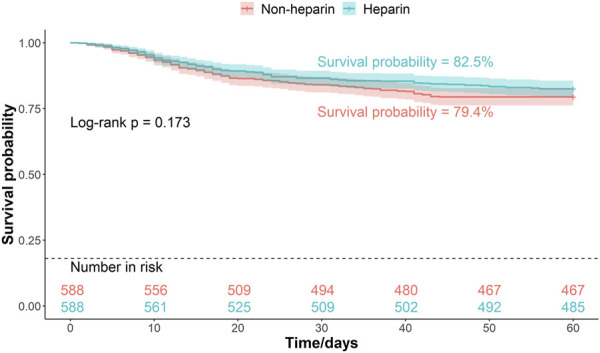
Kaplan-Meier survival curves between the two groups indicating 60-day mortality risk in patients with pneumonia sepsis. (After PSM).

Patients were divided into two groups based on whether they received heparin during hospitalization. The Kaplan–Meier curves of the 30-, 45-, and 60-d survival rates are shown in Figures 2–4, respectively. According to the Kaplan–Meier survival analysis, the 45-d survival rate was significantly higher in the heparin user group than that in the non-user group (log-rank test, P < 0.05). However, the 30- and 60-d survival rates did not differ significantly between the two groups (log-rank test, P > 0.05).

Univariate Cox proportional hazards model revealed that the 45-d mortality risk was lower in the heparin group than that in the non-user group (HR = 0.75, P = 0.034) (Table 3). The association between heparin use and mortality in patients with pneumonia-sepsis was further analyzed using Cox regression models, considering the effects of age and sex. Multivariate Cox proportional hazard analysis confirmed that the 45-d mortality risk remained lower in the heparin group than that non-users (adjusted HR = 0.73, P = 0.025) (Table 3).

**TABLE 3:** 45-day survival outcomes for heparin users and non-users. (After PSM).

	Group	No. events/No. All patients	Log rank test value	Hazard ratio (95%CI)	Adjusted hazard ratio (95%CI)
45-day mortality	Non-heparin	121/588	0.033	HR = 0.75 (0.572–0.983) P = 0.034	Adjusted HR = 0.73 (0.563–0.964) P = 0.025
	Heparin	92/588			

In the 45-d survival analysis of patients with pneumonia-induced sepsis based on heparin dosage, univariate and multivariate Cox regression models were applied. The 0 mL heparin group served as the control. The HR for the 2 mL dose group ranged from 0.794 to 0.809, with p-values ranging from 0.264 to 0.316, indicating no statistically significant effect on survival. The HR in the 3 mL dose group ranged from 0.657 to 0.688, with p-values ranging from 0.010 to 0.023, which were statistically significant, indicating that this dose had a positive effect on patient survival. The 4.5 mL dose group showed an increased mortality risk. The unadjusted model had an HR of 1.925 (P = 0.058), which was not statistically significant. However, after adjusting for factors, such as age and sex, the HR increased to 2.004, (P = 0.049), suggesting a possible increased risk of death at higher doses (Table 4). The effect of heparin use duration on 45-d survival in patients with pneumonia-induced sepsis was also analyzed using univariate and multivariate Cox regression models. The non-heparin group served as the control. Patients treated with heparin for 1–3 d showed no significant survival advantage, with HR values ranging from 0.829 to 0.944 and p-values from 0.379 to 0.789. Similarly, patients who received heparin for 3–7 d showed no statistically significant difference in survival, with HR values ranging from 0.776 to 0.800 and p-values from 0.240 to 0.317. However, patients who received heparin for more than 7 d demonstrated significantly improved survival, with HR values ranging from 0.681 to 0.674 and p-values from 0.026 to 0.037, indicating that prolonged heparin use may have a positive effect on survival (Table 5). These findings suggest that both a moderate dose (3 mL) and extended use (>7 d) of heparin significantly improved the 45-d survival rate of patients with pneumonia-induced sepsis, while higher doses (4.5 mL) were associated with an increased risk of death.

**TABLE 4:** Univariate and multivariate Cox regression models of heparin dosage on 45-day survival in patients with pneumonia-induced sepsis (After PSM).

Variables	Model1		Model2		Model3	
	HR (95%CI)	P	HR (95%CI)	P	HR (95%CI)	P
Heparin Dosage						
0 mL	1.000 (Reference)		1.000 (Reference)		1.000 (Reference)	
2 mL	0.794 (0.529∼1.191)	0.264	0.801 (0.529∼1.213)	0.295	0.809 (0.535∼1.225)	0.316
3 mL	0.657 (0.477∼0.906)	0.010	0.680 (0.493∼0.939)	0.019	0.688 (0.498∼0.949)	0.023
4.5 mL	1.925 (0.978∼3.789)	0.058	2.166 (1.088∼4.310)	0.028	2.004 (1.003∼4.003)	0.049

HR, hazard ratio; CI, confidence interval.

Model 1: Crude.

Model 2: Adjust: Age, Gender.

Model 3: Adjust: Age, Gender, SOFA, diabetes, ARF, COPD, hypertension; AKI, stage.

**TABLE 5:** Univariate and multivariate Cox regression models of heparin duration on 45-day survival in patients with pneumonia-induced sepsis (After PSM).

Variables	Model1		Model2		Model3	
	HR (95%CI)	*P*	HR (95%CI)	*P*	HR (95%CI)	*P*
Duration						
None	1.000 (Reference)		1.000 (Reference)		1.000 (Reference)	
≥1d; ≤3d	0.829 (0.546∼1.258)	0.379	0.941 (0.619∼1.432)	0.777	0.944 (0.621∼1.436)	0.789
>3d; ≤7d	0.776 (0.508∼1.185)	0.240	0.802 (0.521∼1.235)	0.317	0.800 (0.519∼1.231)	0.309
>7d	0.681 (0.475∼0.977)	0.037	0.662 (0.461∼0.952)	0.026	0.674 (0.469∼0.969)	0.033

HR, hazard ratio; CI, confidence interval.

Model1: Crude.

Model2: Adjust: Age, Gender.

Model3: Adjust: Age, Gender, SOFA, diabetes, ARF, COPD, hypertension; AKI, stage.

### 3.3 Association of heparin with secondary outcomes

In-hospital mortality did not differ significantly between the heparin and non-heparin groups (P > 0.05). However, the heparin group had shorter ICU and hospital stays, with no significant difference in gastrointestinal bleeding rates between the two groups (P > 0.05) (Table 6).

**TABLE 6:** Secondary outcomes of the heparin use group and the non-users group. (After PSM).

Secondary outcomes	Overall	Non-heparin	Heparin	P
Los_Hospital (d)	15.94 [8.94, 25.58]	16.96 [10.00, 27.63]	14.56 [7.83, 23.08]	<0.001
Los_ICU (d)	8.55 [3.40, 16.29]	9.39 [4.25, 17.11]	7.72 [2.84, 14.87]	<0.001
Hospital Expire Flag, n (%)	173 (14.71)	94 (15.99)	79 (13.44)	0.217
Gastrointestinal Bleeding (%)	3 (0.3)	2 (0.3)	1 (0.2)	1

### 3.4 Subgroup analyses

**FIGURE 5:**
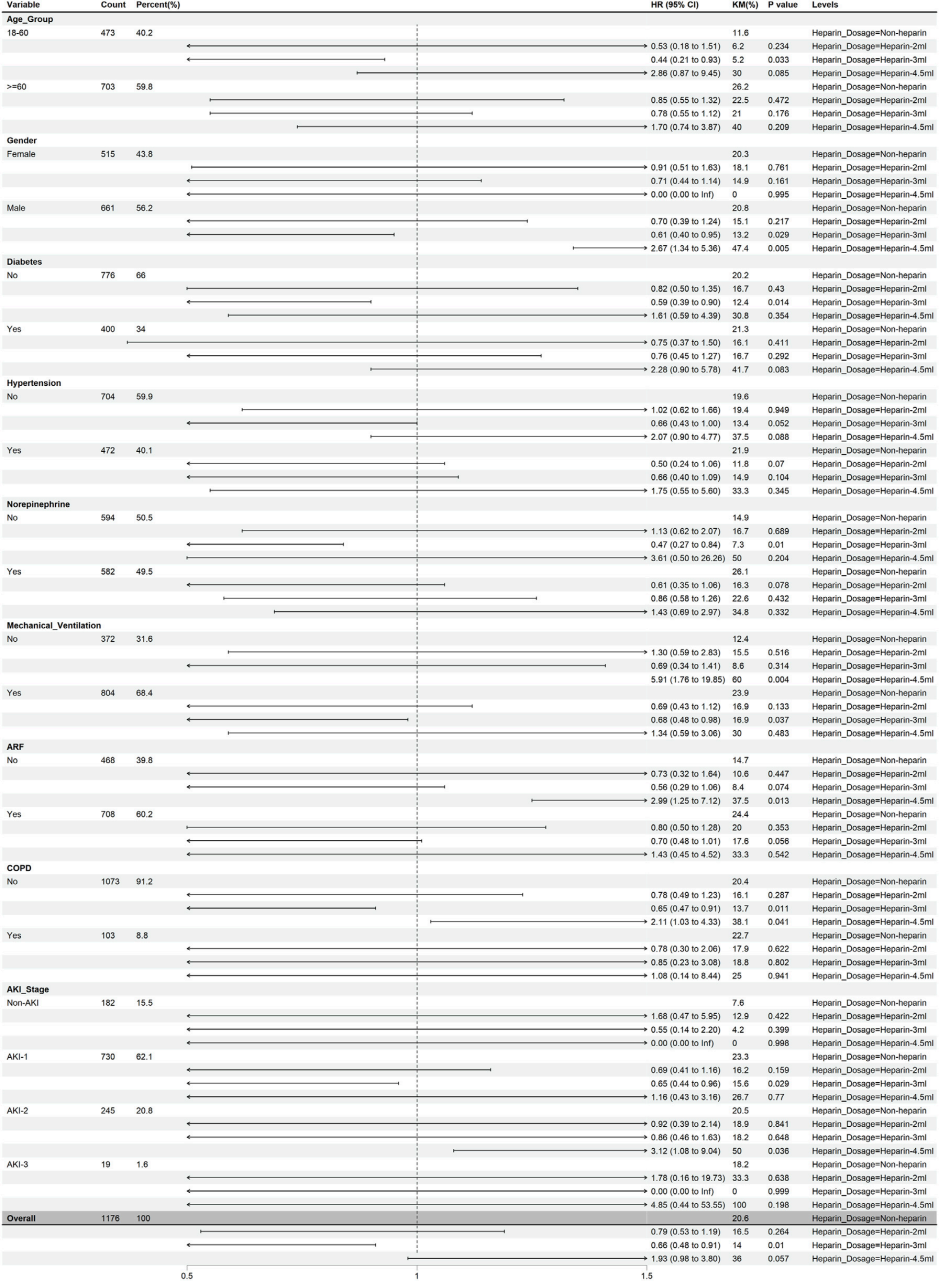
The effects of heparin dose on 45-day mortality in different subgroups. (After PSM).

**FIGURE 6:**
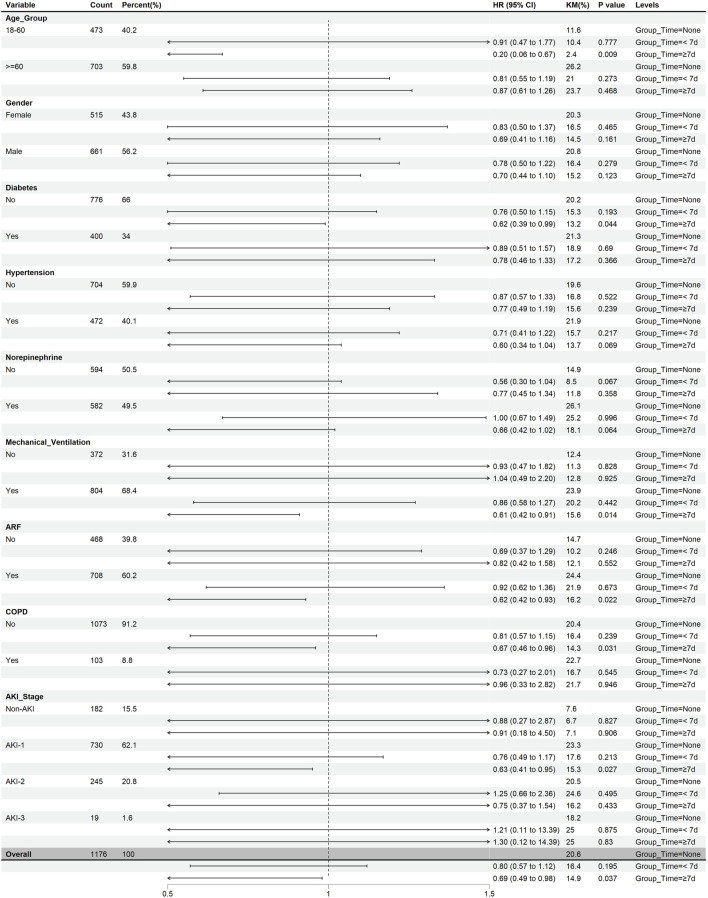
The effect of heparin duration on 45-day mortality in different subgroups. (After PSM).

Patients with pneumonia-induced sepsis were divided into subgroups according to age, sex, ICU life support methods (mechanical ventilation and vasoactive drugs), chronic diseases (diabetes, hypertension, and COPD), and complications (ARF and AKI). Figure 5 shows the effect of heparin dose on 45-d mortality across these subgroups, while Figure 6 illustrates the impact of heparin duration on 45-d mortality across these subgroups.

As shown in Figure 5, using the non-heparin group as a reference, a daily heparin dose of 2 mL did not significantly correlate with 45-d mortality in all subgroups (P > 0.05). A daily heparin dose of 3 mL was associated with reduced 45-d mortality in the following subgroups: age 18–60 years, male, no diabetes mellitus, no norepinephrine use, mechanical ventilation, no COPD, and stage 1 AKI (P < 0.05). Overall, patients treated with heparin had a lower HR, especially at the 3 mL dose, indicating a significant survival advantage (HR = 0.66, 95% CI 0.48–0.91, P < 0.05). At a daily dose of 4.5 mL, heparin was associated with a higher 45-d mortality in the following subgroups: men, no mechanical ventilation, no combined ARF, no combined COPD, and stage 2 AKI (P < 0.05). However, overall P-values did not show significant differences (P > 0.05).

As shown in Figure 6, using the non-heparin group as a reference, no significant correlation was found between heparin use for less than 7 d and 45-d mortality across all subgroups (P > 0.05). When heparin was used for more than 7 d, it was associated with lower 45-d mortality in the following subgroups: age 18–60 years, no diabetes mellitus, mechanical ventilation, ARF, no COPD, and stage 1 AKI (P < 0.05). Overall, patients using heparin had a lower HR, particularly after 7 d of use, showing a significant survival advantage (HR = 0.69, 95% CI 0.49–0.98, P < 0.05).

## 4 Discussion

To the best of our knowledge, this is the first study to demonstrate that heparin administration improved the 45-d survival rate and reduced LOS_ICU in patients with pneumonia-induced sepsis. Based on our findings, we recommend using heparin for more than 7 d at a daily dosage of 3 mL (heparin sodium 5000 units/mL-1 mL vial; TID). Additionally, no significant difference in gastrointestinal bleeding was observed between the heparin and non-heparin groups.

Heparin is a natural glycosaminoglycan produced by mast cells and basophils. It was discovered by McLean in 1916 and was first used in clinical practice as an anticoagulant in 1935 (Barrowcliffe, 2012). Recent studies have shown that heparin possesses additional antitumor, anti-inflammatory, and anti-infective properties (Oduah et al., 2016). UFH is a minimally processed form of naturally occurring heparin, purified from porcine intestines or, less commonly, bovine intestines (Bianchini et al., 1997). The primary anticoagulant effect of UFH is mediated by its binding and activation of two key plasma serpins, antithrombin (AT) and HCII. A specific pentasaccharide sequence with 3-O-sulfated glucosamine residues binds to AT with high affinity, which is a crucial structural feature of UFH’s anticoagulant activity (Hogwood et al., 2017).

UFH may enhance survival in pneumonia-induced sepsis through multiple potential mechanisms. Its well-established antithrombotic properties may play a crucial role in mitigating the thrombotic complications commonly observed in sepsis. Warkentin highlighted that heparin’s ability to prevent microthrombus formation may reduce the severity of organ dysfunction, which is a major contributor to sepsis-related mortality (Warkentin, 2012). Moreover, Rabenstein (Rabenstein, 2002) and Stark & Massberg (Stark and Massberg, 2021) suggested that UFH may counteract the pro-coagulant state in sepsis by inhibiting platelet aggregation and thrombin generation, which are key factors in the pathogenesis of septic shock.

In addition to its antithrombotic effects, heparin also has anti-inflammatory properties that may be beneficial in sepsis management. Fullerton & Gilroy (Fullerton and Gilroy, 2016) emphasized that UFH can modulate inflammation, potentially by regulating the activity of various cytokines and inflammatory mediators. For instance, UFH’s interaction with endothelial cells, as discussed by Joffre et al. (Joffre et al., 2020), may help attenuate endothelial activation, a key feature of sepsis. Heparin has also been shown to reduce the release of HMGB1, a potent mediator of inflammation and immune response, as demonstrated by Lu et al. (2014) and Ni et al. (2020), thus potentially alleviating the exaggerated inflammatory response characteristic of sepsis.

Additionally, UFH may influence immune cell functions, such as the activation of protein C, which has been shown to have both anticoagulant and anti-inflammatory effects (Faioni et al., 2015). This dual mechanism may contribute to improved survival by addressing both the inflammatory and coagulatory aspects of sepsis. Moreover, UFH’s ability to modulate the balance between pro-inflammatory and anti-inflammatory pathways may further enhance its therapeutic potential in sepsis management. Its combined antithrombotic and anti-inflammatory mechanisms support its potential as a therapeutic agent for improving survival in pneumonia-induced sepsis. Further clinical investigations are needed to fully elucidate and confirm these effects in sepsis treatment.

One of the key components of the vascular endothelium is the glycocalyx, which regulates the microvascular environment, coagulation, thrombosis, and vascular permeability. It is a negatively charged network composed of core proteins and side chains (Rizzo and Dudek, 2017). Intravascular glycocalyx contains heparan sulfate, a small fraction of which has a unique pentasaccharide pattern with a strong affinity for plasma AT. The combination of heparan sulfate and AT prevents thrombosis in the microvascular network and helps maintain open microvessels (Shworak et al., 2010).

Vink et al. reported that specific disruption of the glycocalyx accelerates thrombin production and increases platelet adhesion within a short period (Vink et al., 2000). Levels of human polyligand proteoglycan 1 (syndecan-1), a degradation product of the glycocalyx, are associated with disease severity, mortality, and the development of DIC in patients with sepsis (Ikeda et al., 2018). In a study on heparin and lipopolysaccharide (LPS)-induced endothelial barrier dysfunction, UFH was shown to reduce LPS-induced stress fiber and intracellular space formation and decrease the expression of the 225-kDa cytoplasmic phosphoprotein zona occludens (ZO-1). These findings suggest that UFH may enhance endothelial barrier function and reduce vascular leakage by protecting the vascular endothelium (Li et al., 2012).

In an animal study, UFH treatment significantly reduced the levels of inflammatory biomarker syndecan-1 (SDC-1) and heparan sulfate in LPS-induced sepsis rat models. This treatment also reduced the levels of prothrombin fragment 1 + 2, thrombin-AT complex, and plasminogen activator inhibitor-1, suggesting that UFH reduced glycocalyx degradation and sepsis-associated coagulation activation (Huang et al., 2020). Another study confirmed that UFH treatment may reduce pulmonary coagulation activation and inflammation by decreasing histone-induced reduction of lung syndecan-1 mRNA and protein levels, thereby inhibiting lung glycocalyx degradation (Fu et al., 2022).

Thus, the endothelial glycocalyx may serve as a bridge between sepsis-induced coagulation and inflammation. By reducing glycocalyx degradation, UFH effectively maintains endothelial function and stabilizes the vasculature, thereby mitigating the coagulation and inflammatory responses associated with sepsis.

Clinical evidence regarding the use of heparin in sepsis remains inconsistent, with notable variations in treatment practices across institutions and patient populations. This heterogeneity is partly due to selection bias, complicating the interpretation of studies. For example, Jiang et al. (2024) suggested that heparin may reduce mortality in septic patients; however, variations in dosing and treatment duration persist due to the absence of standardized protocols. Yu et al. (2024) also highlighted the lack of guidelines, leading to inconsistent clinical practices. Additionally, Fan et al. (2016) reported substantial variability in outcomes across trials, likely reflecting differences in institutional practices rather than the drug’s efficacy. The role of heparin in sepsis is further complicated by its effects beyond anticoagulation (Li and Ma, 2017).

Safety concerns are also significant, particularly in older patients or those with comorbidities. Studies by Bonfim et al. (2023) and Hogwood et al. (2023) have emphasized the potential for adverse effects, such as bleeding complications, due to improper dosing, underscoring the need for careful monitoring. Similarly, Totoki et al. (2024) noted that while heparin may reduce mortality and organ dysfunction, the risks in high-risk populations—such as the older or those with liver or kidney dysfunction—must be carefully considered. Further research is needed to better understand the safety and optimal use of heparin in these patients.

This study first excluded highly correlated variables using VIF and reduced selection bias through PSM analysis. We found that early heparin use was strongly associated with 45-d mortality in patients with pneumonia-sepsis. Adjusted Cox proportional hazard models indicated that a daily dose of 3 mL heparin administered for more than seven consecutive days had a protective effect on 45-d mortality in these patients. These findings align with those of an open-label, adaptive, multi-platform randomized clinical trial (Investigators et al., 2021), which demonstrated increased survival at discharge and more days without respiratory support with heparin treatment in critically ill patients with sepsis-associated COVID-19. Furthermore, a national cohort study (Rentsch et al., 2021) found that early prophylactic anticoagulation in COVID-19 patients was associated with a reduced risk of 30-d mortality.

In a subgroup analysis, our study found that a daily heparin dose of 3 mL for more than 7 d provided a significant survival advantage in the following subgroups: patients aged 18–60 years, those without coexisting diabetes, COPD, or stage 1 AKI. Heparin alleviates the renal inflammatory response induced by sepsis, improves renal and lung function (Liu et al., 2019; Huang and Kong, 2020), and may benefit patients with sepsis experiencing DIC (Liu et al., 2014), mechanical ventilation use, and shock (Zarychanski et al., 2008). In our study, no significant difference was observed in gastrointestinal bleeding between the heparin and non-heparin groups. Therefore, we suggest that appropriate heparin administration in the early stages of microcirculatory disorders may reduce glycocalyx shedding, prevent further endothelial cell damage, reduce microthrombus formation, and improve near-term survival in patients with pneumonia-sepsis.

This study has some limitations. First, its retrospective design may introduce measurement bias, despite the use of PSM to reduce selection bias. Future prospective, multicenter randomized controlled trials are needed to confirm these findings. Second, missing inflammatory markers, such as C-reactive protein and interleukins, limited the ability to assess heparin’s anti-inflammatory effects. Future studies should include these markers. Finally, the study’s generalizability is limited due to the use of data from a single center. Research involving diverse populations and multiple institutions is needed to enhance external validity.

## 5 Conclusion

Our study suggests that early heparin use may be associated with a reduced risk-adjusted 45-d mortality in patients with pneumonia-sepsis and a decreased overall hospital and LOS_ICU. Additionally, early heparin use did not increase the risk of gastrointestinal bleeding, suggesting that it may be a safe and effective therapeutic option for this patient population. A daily dose of 1 mL per dose, TID, administered for more than 7 days was associated with a significant survival advantage in patients with pneumonia-sepsis, particularly in the following subgroups: patients aged 18–60 years, those without coexisting diabetes, COPD, or stage 1 AKI. These findings support the potential role of early heparin therapy as part of the management strategy for pneumonia-induced sepsis. Future prospective studies are warranted to confirm these results and explore the underlying mechanisms of heparin’s effects in sepsis treatment.

## Data Availability

Publicly available datasets were analyzed in this study. This data can be found here: https://physionet.org/content/mimiciv/3.0/ (certification number: 63162441).
